# Strand-specific PCR of UV radiation-damaged genomic DNA revealed an essential role of DNA-PKcs in the transcription-coupled repair

**DOI:** 10.1186/1471-2091-12-2

**Published:** 2011-01-08

**Authors:** Jing An, Tianyi Yang, Yuecheng Huang, Feng Liu, Jingfen Sun, Yu Wang, Qingzhi Xu, Dechang Wu, Pingkun Zhou

**Affiliations:** 1 Department of Radiation Toxicology and Oncology, Beijing Institute of Radiation Medicine, 27 Taiping Road, Beijing 100850, P. R. China; 2 The Institute of Environmental Pollution and Health, School of Environmental and Chemical Engineering, Shanghai University, Shanghai, 200444, P. R. China; 3 Department of Radiation Medicine, the Second Military Medical University, PLA China, Shanghai, 200433, P. R. China

## Abstract

**Background:**

In eukaryotic cells, there are two sub-pathways of nucleotide excision repair (NER), the global genome (gg) NER and the transcription-coupled repair (TCR). TCR can preferentially remove the bulky DNA lesions located at the transcribed strand of a transcriptional active gene more rapidly than those at the untranscribed strand or overall genomic DNA. This strand-specific repair in a suitable restriction fragment is usually determined by alkaline gel electrophoresis followed by Southern blotting transfer and hybridization with an indirect end-labeled single-stranded probe. Here we describe a new method of TCR assay based on strand-specific-PCR (SS-PCR). Using this method, we have investigated the role of DNA-dependent protein kinase catalytic subunit (DNA-PKcs), a member of the phosphatidylinositol 3-kinase-related protein kinases (PIKK) family, in the TCR pathway of UV-induced DNA damage.

**Results:**

Although depletion of DNA-PKcs sensitized HeLa cells to UV radiation, it did not affect the ggNER efficiency of UV-induced cyclobutane pyrimidine dimers (CPD) damage. We postulated that DNA-PKcs may involve in the TCR process. To test this hypothesis, we have firstly developed a novel method of TCR assay based on the strand-specific PCR technology with a set of smart primers, which allows the strand-specific amplification of a restricted gene fragment of UV radiation-damaged genomic DNA in mammalian cells. Using this new method, we confirmed that siRNA-mediated downregulation of Cockayne syndrome B resulted in a deficiency of TCR of the UV-damaged dihydrofolate reductase (*DHFR*) gene. In addition, DMSO-induced silencing of the c-myc gene led to a decreased TCR efficiency of UV radiation-damaged c-myc gene in HL60 cells. On the basis of the above methodology verification, we found that the depletion of DNA-PKcs mediated by siRNA significantly decreased the TCR capacity of repairing the UV-induced CPDs damage in *DHFR *gene in HeLa cells, indicating that DNA-PKcs may also be involved in the TCR pathway of DNA damage repair. By means of immunoprecipitation and MALDI-TOF-Mass spectrometric analysis, we have revealed the interaction of DNA-PKcs and cyclin T2, which is a subunit of the human transcription elongation factor (P-TEFb). While the P-TEFb complex can phosphorylate the serine 2 of the carboxyl-terminal domain (CTD) of RNA polymerase II and promote transcription elongation.

**Conclusion:**

A new method of TCR assay was developed based the strand-specific-PCR (SS-PCR). Our data suggest that DNA-PKcs plays a role in the TCR pathway of UV-damaged DNA. One possible mechanistic hypothesis is that DNA-PKcs may function through associating with CyclinT2/CDK9 (P-TEFb) to modulate the activity of RNA Pol II, which has already been identified as a key molecule recognizing and initializing TCR.

## Background

Cellular genomic DNA constantly suffers from damage induced by various external genotoxic agents and endogenous metabolic materials. In eukaryotic cells there are multiple conserved DNA repair pathways including nucleotide excision repair (NER), which is a DNA repair mechanism removing a variety of helix-distorting DNA lesions including ultraviolet radiation (UV)-induced cyclobutane pyrimidine dimers (CPD), 6-4 pyrimidine pyrimidone photoproducts [(6-4)PPs], and cigarette smoke-induced benzo[a]pyrene DNA adducts. The relevance of this repair pathway is indicated by the observation that defected NER genes can result in rare human autosomal recessive disorders such as xeroderma pigmentosum (XP) and Cockayne syndrome (CS) [1]. There are two NER sub-pathways: global genomic NER repair (ggNER) and transcription-coupled repair (TCR), which differ mainly in the step of recognition of the DNA lesions [1,2]. TCR preferentially repairs the transcribed strand or transcribed genes compared to the untranscribed strand or silenced genes. In other words, the transcribed strand or genes that are undergoing transcription exhibit a faster rate of repairing DNA damage than the untranscribed strand and the overall genome [3-6]. RNA polymerase II plays a critical role in the recognition of DNA damage in the TCR pathway. The current TCR model proposes that RNA polymerase, stalled at a lesion point, directs the recruitment of repair enzymes to the transcribed strand of an active gene [7-10]. This model assumes that RNA polymerase II must be removed from the lesion site of the transcribed strand to provide access for the repair complex, which initiates the repair process through unwinding the double helix at the damaged site, removal of the DNA terminus, and finally filling the gap and joining the DNA strands. Previous studies have shown that TCR is a critical survival pathway protecting against acute toxic and long-term effects of genotoxic exposures [11]. A number of human genetic syndromes such as Xeroderma pigmentosum complementing group D and Cockayne syndromes A and B (CSA, CSB) have been identified as associated with a deficient TCR mechanism against UV radiation, and therefore the cells from these patients are sensitive to UV irradiation [1,2,4,12]. In recent years, extensive knowledge regarding TCR has been acquired. However, as mentioned by the TCR research pioneers, Hanawalt and Spivak, the more we have learned, the more questions have been raised about the intricate details of TCR and its relevance to human disease [2].

DNA dependent protein kinase catalytic subunit (DNA-PKcs) is a member of the phosphatidylinositol 3-kinase-related protein kinase (PIKK) family with serine/threonine kinase activity, and it is well known as a critical component of the non-homologous end joining (NHEJ) pathway of DNA double-strand break (DSB) repair [13]. When DSB occurs in the cells, the Ku80/Ku70 heterodimer firstly recognizes and binds to the broken DNA, followed by recruitment of DNA-PKcs to the damage site to form the DNA-PK complex with Ku80/Ku70, which consequently triggers the damage signal and subsequent DNA repair [14]. It has previously been reported that cells with mutated or decreased expression of DNA-PKcs are sensitive to UV irradiation [15,16]. Furthermore, mouse SCID cells or hamster V-3 cells with mutated DNA-PKcs exhibit a decreased capability of NER activity, with reduced unscheduled DNA synthesis (UDS) after UV irradiation [16]. However, this NER deficiency cannot be complemented by addition of DNA-PK, suggesting a non-direct role of DNA-PK in the NER repair steps. Since RNA polymerase II is a substrate of DNA-PKcs [17], it was suggested that DNA-PK could act as a DNA damage-sensor that might regulate DNA repair via transcriptional modulation [16].

In this study, we postulated that DNA-PKcs might play a role in the transcription-coupled repair pathway of UV-induced CPDs damage. In order to detect the TCR of ultraviolet (UV) radiation-induced DNA damage, we established a novel method of TCR assay based on DNA strand-specific PCR (SS-PCR) of the dihydrofolate reductase (*DHFR*) gene. We verified the efficiency and feasibility of this TCR assay in a siRNA-mediated CSB-depleted cell model, in which CSB depletion resulted in a deficient TCR pathway. In addition, TCR of the UV damaged c-myc gene was also found to be attenuated when the c-myc gene was silenced by the treatment of DMSO in HL60 cells. Using this verified method, we demonstrated that siRNA-mediated silencing of DNA-PKcs has no effect on the ggNER efficiency, while repair efficiency of that transcribed strand (but not the non-transcribed strand) of *DHFR *gene was significantly lower in DNA-PKcs-depleted HeLa-H1 cells than that in control HeLa-NC cells. Furthermore, we have carried out the co-immunoprecipitation assay and identified an interaction between DNA-PKcs and the cyclin T2 protein. CyclinT2, together with Cyclin-dependent kinase 9 (CDK9), comprises the positive transcription elongation factor b (P-TEFb). The activated CDK9 phosphorylates the carboxyl terminal domain (CTD) of RNA Pol II, and then promotes transcription elongation [18,19]. Hence, we postulate that DNA-PKcs may modulate the activity of RNA Pol II through associating with cyclin T2. The present study provides new evidence suggesting the involvement of DNA-PKcs in the transcription-coupled repair pathway of nucleotide excision repair in response to UV-induced DNA damage.

## Results

### Depletion of DNA-PKcs enhanced the cellular UV sensitivity

**Figure 1:**
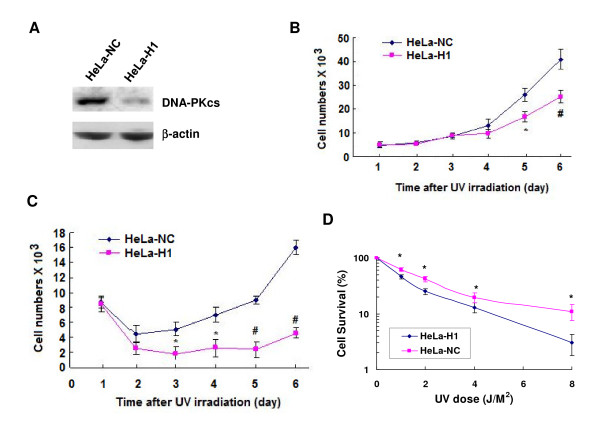
**Depletion of DNA-PKcs by specific siRNA sensitizes cells to UV irradiation**. A: Immunoblotting analysis shows downregulated expression of DNA-PKcs in HeLa-H1 as compared with the control HeLa-NC cells. The HeLa-H1 and HeLa-NC cells were generated from HeLa cells by stably transfecting with a shRNA vector expressing siRNA specifically targeting DNA-PKcs, and a control siRNA vector expressing a molecule showing no homology with the mammalian genomic DNA. B: Cell growth curves after exposed to 5 J/m^2 ^of Ultraviolet (UV). The data are the means of four independent experiments with standard deviation. * *P *< 0.05, # *P *< 0.01 as compared with HeLa-NC cells at the same time point. C: Cell growth curves after exposed to 10 J/m^2 ^of Ultraviolet (UV). The data are the means of four independent experiments with standard deviation. * *P *< 0.05, # *P *< 0.01 as compared with HeLa-NC cells at the same time point. D: Cell survival (clonogenic assay) after exposed to different dose of UV radiation. The data are the means of three independent experiments with standard deviation. * *P *< 0.05 as compared with HeLa-NC cells at the same UV dose.

As shown in the growth curve (Figure 1), depletion of DNA-PKcs mediated by transfecting with the shRNA vector expressing the specific siRNA molecules targeting the catalytic motif of the DNA-PKcs gene (nucleotides 11637~11655, H1) significantly decreased the growth ability of HeLa-H1 as compared to the control HeLa-NC cells after exposure to 5 J/m^2 ^(Figure 1B) and 10 J/m^2 ^(Figure 1C) UV irradiation. To confirm these results, a clonogenic assay was performed, and the cell survival levels also showed an increased sensitivity of the DNA-PKcs-depleted HeLa-H1 cells to UV radiation as compared with the control HeLa-NC cells (Figure 1D).

### Depletion of DNA-PKcs does not affect the ggNER of UV-induced DNA damage

**Figure 2:**
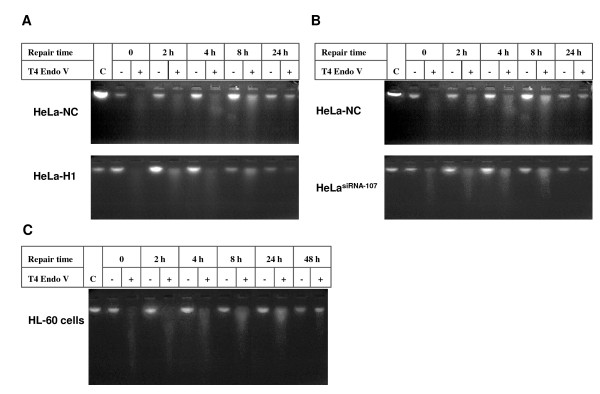
**The global genomic nucleotide excision repair (ggNER) detected using alkaline electrophoresis**. A: Representative alkaline electrophoresis pattern in the ggNER assay of DNA-PKcs depleted HeLa-H1 cells and control HeLa-NC cells. Cells were collected at 0, 2, 4, 8 and 24 h after 10 J/m^2 ^UV radiation, then genomic DNA was digested with T4 Endo V, and ggNER was assayed using alkaline electrophoresis. Genomic DNA without T4 Endo V treatment at the same time point was used as the control. B: A representative alkaline electrophoresis pattern in the ggNER assay of CSB depleted HeLa^siRNA-107 ^cells and control HeLa-NC cells. HeLa^siRNA-107 ^cells were generated from HeLa cells by stably transfecting with a vector expressing siRNA specifically targeting the *CSB *gene. C: A representative alkaline electrophoresis pattern in the ggNER assay of HL60 cells.

DNA damage from UV exposure results in the frequent formation of cyclobutane pyrimidine dimers (CPD) and 6-4 pyrimidine pyrimidone photoproducts [(6-4) PPs]. The thymine dimer in DNA sequences can be effectively recognized and incised by T4 Endo V. With increased repair time, the residual thymine dimers decrease, and the extent of fragmentation of genomic DNA by T4 Endo V is also decreased. Therefore, detection of genomic fragmentation by the alkaline electrophoresis assay can indicate the whole genomic DNA repair efficiency. As shown in Figure 2, the global genome nucleotide excision repair (ggNER) was completed 24 h after UV irradiation, and there was no difference in ggNER efficiency between DNA-PKcs-depleted cells (HeLa-H1) and control cells (HeLa-NC) after 10 J/m^2 ^UV exposure (Figure 2A). We also compared the ggNER efficiency in HeLa cells possessing a decreased expression of the *CSB *gene. As shown in Figure 2B, depression of the *CSB *expression mediated by siRNA also had no obvious effect on ggNER in HeLa^siRNA-107 ^cells as compared to the control HeLa-NC cells.

The HL60 cell line is sensitive to UV radiation, and the alkaline electrophoresis result confirmed that the ggNER efficiency of UV-induced DNA damage in HL60 cells is slower than in HeLa cells (Figure 2C). Although the DNA damage was almost completely repaired at 24 h after 10 J/m^2 ^UV radiation in HeLa cells (Figure 2A &2B), that was not accomplished in HL60 cells until 48 h after exposure (Figure 2C).

### Development and validation of the novel method of TCR assay based on DNA strand-specific PCR

**Figure 3:**
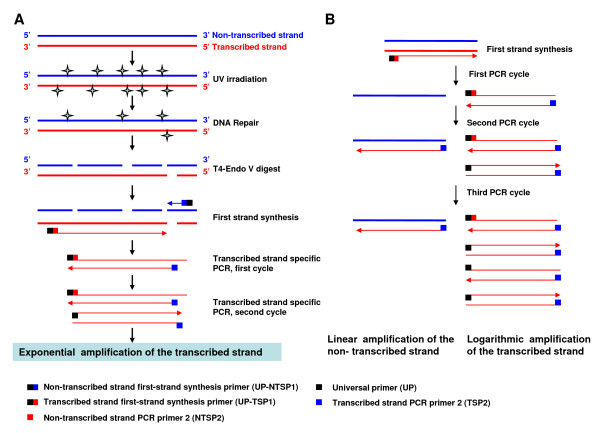
**The procedure outline of the novel method of transcription-coupling repair (TCR) assay based on strand-specific polymerase chain reaction (SS-PCR)**.

Although depletion of DNA-PKcs sensitizes HeLa cells to UV radiation, our results show no obvious effect on the efficiency of the ggNER activity. Next, we decided to focus on the transcription-coupled repair (TCR) of UV-induced DNA damage. The available classical method of TCR assay is time-consuming, requires running sequencing gels, and uses radioactive isotopic probes in southern blotting hybridization[20,21]. Here we designed a novel TCR assay (Figure 3A) based on strand-specific polymerase chain reaction (SS-PCR) with a set of smartly designed primers (Table 1). This method partially evolved from the idea of the quantitative PCR detection of transcription-coupled repair based on the chromatin co-immunoprecipitated DNA fragments [22], and the primers designing strategy of the suppression subtractive hybridization PCR method [23]. The general theoretical basis and the technical principles are as follows: 1) It is based on the DNA strand-specific PCR method to amplify the transcribed strand or untranscribed strand in the separate reaction tube with specifically designed primers for each; 2) The repair efficiency of the transcribed and untranscribed strands is reflected by the PCR efficiency of the synthesized first-strand of the transcribed or untranscribed strand using T4 Endo V-digested genomic DNA as a template, which is directly related to the repair efficiency of CPDs located in DNA strands.

**Table 1:** Primers for the strand-specific PCR and PCR conditions

Aim	Primers	**Primer sequence **^**1**^	PCR condition
First strand synthesis of *DHFR *transcribed strand	DHFR_UP-TSP1	5'-GAGTCGAGTCGACATCGAGCCACTGTGCCCGGTCAGAC-3'	94°C 15 s, 62°C 15 s, 72°C 60 s for 1 cycle

First strand synthesis of *DHFR *non-transcribed strand	DHFR_UP-NTSP1	5'-GAGTCGAGTCGACATCGGGATGTGCCCTGGTGACTGGA-3'	94°C 15 s, 62°C 15 s, 72°C 60 s for 1 cycle

Amplification of *DHFR *transcribed strand	DHFR_TSP2	5'-GGATGTGCCCTGGTGACTGGA-3'	94°C 15 s, 62°C 15 s, 72°C 40 s for 25 cycles
		
	UP	5'-GAGTCGAGTCGACATCG-3'	

Amplification of *DHFR *no-transcribed strand	DHFR_NTSP2	5'-AGCCACTGTGCCCGGTCAGAC-3'	94°C 15 s, 62°C 15 s, 72°C 40 s for 25 cycles.
		
	UP	5'-GAGTCGAGTCGACATCG-3'	

First strand synthesis of *c-myc *transcribed strand	c-myc_UP-TSP1	5'-GAGTCGAGTCGACATCGAACTTTGTGCCTTGGATTT-3'	94°C 15 s, 58°C 15 s, 72°C 60 s for 1 cycle

Amplification of *c-myc *transcribed strand	c-myc_TSP2	5'-TACTGGAGCTGATGCTGAGC-3'	94°C 15 s, 58°C 15 s, 72°C 40 s for 25 cycles
		
	UP	5'-GAGTCGAGTCGACATCG-3'	

Amplification of *β-actin*	β-actin P1	5'-GCCAGGTCCAGACGCAGGAT-3'	94°C 15 s, 62°C 15 s, 72°C 10 s for 22 cycles
		
	β-actin P2	5'-TGCTATCCCTGTACGCCTCTG-3'	

^1 ^The underline sequence is an adaptor universal primer (UP) without homology to mammalian genomic DNA.

The experimental steps of this method are outlined in Figure 3A: 1) Digest the genomic DNA extracted from UV exposed cells with T4 Endo V to nick the DNA at the residual CPDs damage sites; 2) After DNA denaturation, the first-strand DNA is synthesized using the specific first-strand synthesis primer (UP-TSP1 or UP-NTSP1), which is composed of an upstream adaptor universal primer (UP) and a downstream annealing primer specifically targeting the active transcribed-strand (TSP1) or the untranscribed-strand (NTSP1); 3) DNA elution is done to remove the free first-strand primer, and PCR amplification is carried out using the primer pairs of the UP (universal primer) and the strand-specific down-stream primer targeting the first-strand template of the transcribed strand (TSP2) or the untranscribed strand (NTSP2); 4) Analysis of the PCR products by electrophoresis. The lower efficiency of repairing the damaged DNA strand, the more CPDs damage sites remain. Consequently, more residual damage sites provide more nicking sites for T4 Endo V, which eventually lead to a less amount of intact DNA templates for the first-strand synthesis and the PCR amplification. If both strands of the double-strand DNA molecule is intact (no CPD site in both transcribed- and untranscribed-strand), the transcribed-strand is amplified logarithmically in the transcribed-strand PCR reaction tube due to the smart PCR primer pair of TSP2 and UP which is appended into the templates during the first-strand synthesis of the transcribed-strand. Although TSP2 can also anneal with the complementary untranscribed-strand, it is amplified linearly due to only one primer available for this untranscribed-strand during PCR reaction (Figure 3B). In the same way, the untranscribed-strand is amplified logarithmically and the transcribed-strand is amplified linearly in the untranscribed-strand PCR reaction tube due to the smart PCR primer pair of NTSP2 and UP. Therefore, the transcribed strand or untranscribed strand can be easily determined in this method. Concurrently, a β-actin gene fragment without a TT dimer sequence was used as an internal template reference.

**Figure 4:**
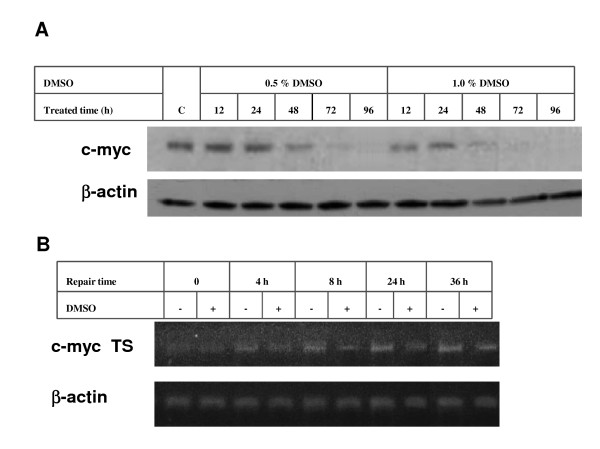
**The influence of gene transcriptional activity on the efficiency of the nucleotide excision repair of DNA damage in the c-myc gene induced by UV radiation**. A: Alteration of c-myc expression in HL60 cells induced by DMSO, detected by immunoblotting analysis. HL60 cells were collected at 12, 24, 48, 72 and 96 h after the treatment with 0.5% or 1% (v/v) of DMSO. Non-DMSO-treated HL60 cells were used as control. B: The influence of gene transcriptional activity on the repair efficiency of DNA damage in the transcribed strand of the c-myc gene, detected by transcribed-strand specific PCR assay. HL60 cells were collected at 0, 4, 8, 24 and 36 h after the treatment with 1% (v/v) DMSO. Non-DMSO-treated HL60 cells were used as the control.

In order to validate the reliability and efficiency of this new method, we firstly detected the TCR efficiency of the c-myc gene in UV-irradiated HL-60 cells under its different transcriptional status. Both mRNA and protein levels of c-myc are overexpressed in HL60 cells. When HL60 cells are induced to differentiate by DMSO, the transcriptional activity of c-myc is rapidly silenced[21]. As shown in Figure 4A, c-myc expression in HL60 cells is remarkably silenced after the treatment of DMSO at 0.5~1% (v/v) and c-myc expression is almost completely inhibited after 1% (v/v) DMSO treatment for 48-72 h. For the following experiments, HL60 cells were treated with 1% DMSO for 72 h, and then irradiated with 10 J/m^2 ^UV. The TCR efficiency of c-myc was then detected using this new method. The results showed that the repair efficiency of the UV-damaged c-myc transcribed strand in DMSO treated cells was significantly decreased as compared with the control cells without DMSO treatment (Figure 4B). This result indicates that the new TCR assay method based on strand-specific PCR is reliable for the detection of TCR of DNA damage.

**Figure 5:**
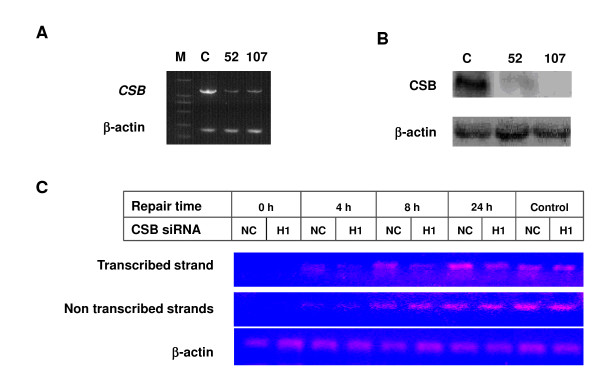
**Transcription coupled repair (TCR) was detected by the strand-specific PCR, and CSB depletion led to decreased efficiency of TCR**. A & B: Transfection of specific siRNA-expressing vectors (CSB^siRNA-52 ^and CSB^siRNA-107^) mediated depression of the *CSB *gene in HeLa cells, detected by semi-quantitative RT-PCR assay (A) and immunoblotting assay (B). C: A representative strand specific PCR to assay TCR in CSB depleted HeLa^siRNA-107 ^and the control HeLa-NC cells. Depression of CSB resulted in a decreased repair efficiency of the *DHFR *gene transcribed-strand DNA damage induced by UV, but there was no influence on the untranscribed strand DNA damage repair. Cells were collected at 0, 2, 4, 8 and 24 h after 10 J/m^2 ^UV radiation, then TCR efficiency was assayed by SS-PCR as described in method.

We then used this new method to further assess the TCR of UV-induced DNA damage in a Cockayne syndrome B gene (*CSB*)-depleted cell line (HeLa^siRNA-107^), which was generated from HeLa cells by transfecting specific siRNA construct to deplete the *CSB *mRNA (Figure 5A) and protein expression (Figure 5B). CSB previously has been shown to play an important role in the TCR pathway. After 10 J/m^2 ^UV radiation, strand-specific PCR of Endo V enzyme digested transcribed and untranscribed strands of *DHFR *gene was performed. As shown in Figure 5C, no difference in PCR efficiency of the untranscribed strand was observed between HeLa^siRNA-107 ^and the control cells, but the PCR efficiency of the transcribed strand in HeLa^siRNA-107 ^cells was obviously lower compared to that in the control cells at all repair time points post UV-irradiation. It seems that the repair was not accomplished even at 24 h post-irradiation in HeLa^siRNA-107 ^cells as compared with control HeLa-NC cells.

### Depletion of DNA-PKcs leads to deficiency of the transcription-coupled repair of UV-induced DNA damage

**Figure 6:**
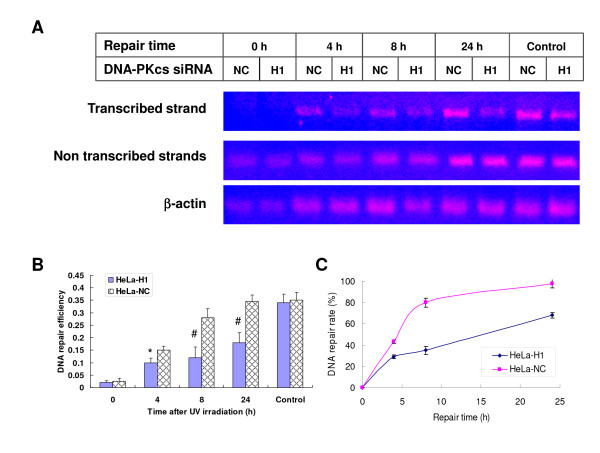
**Transcription coupled repair (TCR) was detected by the strand-specific PCR, and depletion of DNA-PKcs led to decreased efficiency of TCR**. A: A representative strand specific PCR to assay TCR in DNA-PKcs depleted HeLa-H1 and the control HeLa-NC cells. siRNA-mediated depression of DNA-PKcs resulted in a decreased repair efficiency of the *DHFR *gene transcribed-strand DNA damage induced by UV radiation, but there was no influence on the untranscribed strand DNA damage repair. B: Quantitative detection of TCR of the transcribed strand damage in DNA-PKcs depleted HeLa-H1 and control HeLa-NC cells. The DNA repair efficiency is expressed as the ratio of PCR products intensity of the *DHFR *transcribed-strand to β-actin. The data are the means of four independent experiments with standard deviation. * *P *< 0.05, # *P *< 0.01 as compared with HeLa-NC cells at the same time point. C: DNA repair (TCR) rate of the transcribed strand damage in DNA-PKcs depleted HeLa-H1 and control HeLa-NC cells after different time repair post UV irradiation. DNA repair rates were derived from the DNA repair efficiency data in Figure 6B, i.e. the ratio of PCR products intensity of the *DHFR *transcribed-strand to β-actin (ratio of TS to actin). DNA repair rate (%) = (Ratio of TS to actin at a repair time/untreated control) × 100

On the basis of the above observation of DNA damage repair in the transcribed strand of the c-myc gene in DMSO-treated HL60 cells and *DHFR *gene in *CSB*-depleted HeLa^siRNA-107 ^cells, we investigated the effect of DNA-PKcs on the TCR efficiency of UV radiation-damaged DNA using DNA-PKcs-depleted cell line HeLa-H1 [15]. As shown in Figure 6A, no difference in repair efficiency was observed in the untranscribed strand of the *DHFR *gene between DNA-PKcs silenced cells (HeLa-H1) and control cells (HeLa-NC). However, the repair efficiency of the transcribed strand was significantly decreased in HeLa-H1 cells as compared with that in control cells (Figure 6A, B &6C). Approximately 45% of the transcribed strand DNA damage repair was completed at 4 after exposure and 80% of DNA damage was repaired at 8 h in HeLa-NC cells, while the repair kinetics of DNA damage in the transcribed strands was much slower in HeLa-H1 cells (Figure Figure 6B &6C), suggesting a deficiency of TCR in DNA-PKcs-depleted cells.

### The interaction of DNA-PKcs with cyclin T2

**Figure 7:**
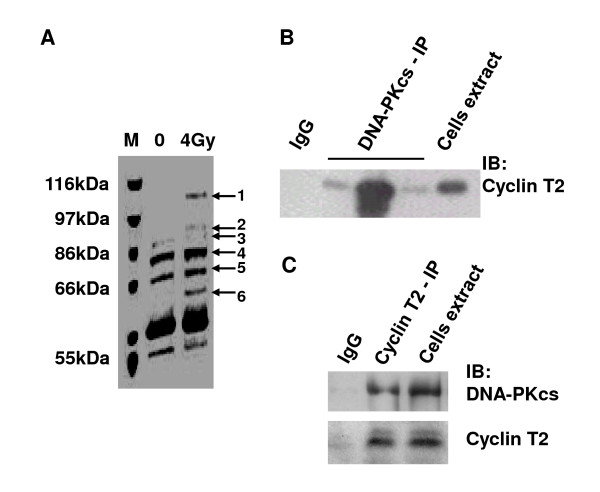
**The interaction of DNA-PKcs with cyclin T2**. A: Coomassie bright blue dye staining of SDS-PAGE of the co-immunoprecipitation products of DNA-PKcs antibody. B: Immunoblotting analysis of cyclin T2 was performed on the CoIP products of DNA-PKcs antibody and total extracts of cells. Three DNA-PKcs lanes represent three independent repeat IP experiments. C: Immunoblotting analysis of DNA-PKcs and cyclin T2 was performed on the CoIP product of cyclin T2 antibody and the total extracts of cells. The immunoprecipitation product of Ig G was taken as the blank control.

Co-immunoprecipitation (CoIP) using a DNA-PKcs antibody was performed in HeLa cell extracts. A number of DNA-PKcs binding proteins were displayed by staining of the immunoprecipitates, resolved by SDS-PAGE, with Coomassie bright blue (Figure 7A). MALDI-TOF-MS analysis indicated that band-5 was cyclin T2 protein (data not shown), revealing the interaction of DNA-PKcs with cyclin T2. Although the 4 Gy γ-ray irradiated cells were used in this assay, an intensive band-5/cyclin T2 has also appeared in the immunoprecipitates from the untreated cells, indicating the interaction of DNA-PKcs and cyclin T2 can occur in the normal growing condition. Therefore, we did not further perform this assay in UV-irradiation cells. The cyclin T2 protein was further detected in the immunoprecipitates of DNA-PKcs antibody by immunoblotting analysis using an antibody against cyclin T2 (Figure 7B). Alternatively, the DNA-PKcs protein has also been detected in the immunoprecipitates of cyclin T2 antibody by immunoblotting analysis using an antibody against DNA-PKcs (Figure 6C).

## Discussion

The nucleotide excision repair (NER) pathway operates through two sub-pathways: global genome repair (ggNER) and transcription-coupled repair (TCR) or gene- and strand-specific DNA repair [1,2,4]. The ggNER is a repair mechanism which has the ability to repair DNA damage to the overall genome with equivalent efficiency. In contrast, TCR is a kind of heterogeneous DNA repair, where repair to the damaged DNA in the status of transcription activity is superior to the silenced genes and the repair of the transcribed strand is superior to the untranscribed strand. Some DNA repair proteins and transcription factors have been identified to be involved in TCR such as CSA, CSB, XPG, XAB2, RNA polymerase II, and TFIIH [1,7,8,24]. Blockage of RNA polymerase □ at the DNA damage site is believed to create a conducive environment for DNA repair [7,9]. In this report, we provide evidence to demonstrate that DNA-PKcs, a known critical component in the NHEJ pathway of DNA double-strand breaks, is also necessary for the TCR pathway of UV radiation-induced DNA damage. We firstly demonstrated that deficient of DNA-PKcs or CSB does not affect the efficiency of the global genome nucleotide excision repair (ggNER). In addition, the ggNER repair kinetics of UV radiation-induced CPDs in HeLa cells (Figure 2A &2B) was shown faster than that in HL60 cells (Figure 2C). A previous report indicated that the level of UVB-induced γH2AX foci (a molecule marker of DNA double-strand break, DSB) in the nonreplicating G1, G2 and M was much higher in HeLa than in HL60 cells[25]. It is well known that the cyclobutane pyrimidine dimers (CPDs) and pyrimidine (6-4) pyrimidone photoproducts (6-4PPs) are the predominant lesions caused by UV radiation. The formation of DSB in the UV-irradiated cells has been explained as resulting from [26,27]: 1) the attempted replication of DNA (S phase cells) at the sites containing the stalling replication forks with these sites attracted an endonuclease which subsequently cleaved DNA generating DSB; 2) the processing of the nucleotide excision repair (NER) of CPDs, during which the endonucleases, *e.g. *ERCC1-XPF, incise the adjacent CPDs in inter-strand of DNA to a DSB. Therefore, the formation of DSB, especially in G1, G2 and M phases, can be considered as the intermediate product of NER, and the increased level of the transient γH2AX foci may reflect a more active NER in HeLa cells than in HL60 cells. At present, we could not give a precise mechanistic explanation on this different kinetics of CPDs repair between HeLa and HL60 cells.

In order to determine the TCR of UV-induced cyclobutane pyrimidine dimers (CPDs), we firstly established a novel method of assaying TCR based on the technology of strand-specific polymerase chain reaction. The classical method of the gene- and strand-specific DNA repair or TCR assay has a tedious procedure including: DNA is firstly cut with appropriate restriction enzymes, and then cut at UV-damaged CPDs with T4-endonuclease V; The digested DNA is electrophoresed in the alkaline agarose gels, blotted to Nylon membranes; Northern hybridization is performed with radioactively labeled strand-specific DNA probes. Obviously, the time consuming and radioactive operation largely limit its application. Up to now, there is still no an ideal alternative method for this purpose. This novel method has solved the major technical obstacles of time consuming and radioactive operation. In principal, it partially evolved from the idea of the quantitative PCR detection of transcription-coupled repair based on the chromatin co-immunoprecipitated DNA fragments [22] and the technique of the tagged RT-PCR for the strand-specific detection of hepatitis C virus (HCV) RNA [28], as well as the primers design strategy of the suppression subtractive hybridization PCR method [23]. This novel method only has the procedures of DNA digestion with T4 Endo V, first-strand synthesis of the single strand and the strand-specific PCR (Figure 3A) with a set of smart specific primers as shown in table 1. In order to insert an exogenous primer into the synthesized first-strand, chimera primer (UP-TSP1 or UP-NTSP1) was used for the first-strand synthesis of transcribed- or untranscribed-strand. The chimera primer is composed of the upstream adaptor universal primer (UP), which has no homology with the genomic DNA of eukaryotic cells, and the downstream annealing primer (TSP1 or NTSP1). As the TT sequence in genes easily can be damaged into TT-dimers by UV irradiation, we selected a TT-rich sequence of *DHFR *as a target to detect the activity of the TCR repair pathway. After exposure to UV radiation, the genomic DNA was digested with T4 Endo V to excise the residual TT dimers, resulting in nicks in the DNA strand, which led to the decrease of template DNA molecules. The first-strands of the transcribed and non-transcribed strands were synthesized separately using the specially designed primers described above. Finally, this synthesized first-strand was used as the template to amplify separately the transcribed and untranscribed strands using the primer pair of an adaptor universal primer (UP) and a primer specifically targeting the *DHFR *gene. This novel method was verified through the detection of the TCR efficiency of the *DHFR *gene in a CSB-depleted cell line (HeLa^siRNA-107^). Our data showed that depletion of CSB led to a decreased efficiency of TCR to repair UV-induced DNA damage in HeLa cells. Using our novel TCR assay, we also confirmed that the TCR efficiency of the c-myc gene was depressed when the c-myc gene transcription was silenced by DMSO treatment in HL60 cells. We found that deficiency of DNA-PKcs does not significantly affect the efficiency of the ggNER activity of UV-damaged genomic DNA. However, we observed that the TCR efficiency of the transcribed strand of the *DHFR *gene in the DNA-PKcs-depleted HeLa-H1 cells was significantly lower than that of control HeLa-NC cells.

The interaction between DNA-PKcs and Cyclin T2 was identified using a co-immunoprecipitation test and a peptide fingerprint assay in this study. Cyclin T2 is a member of the cyclin T family, which is not directly involved in cell cycle control, but involved in the regulation of the gene transcription process through forming the human transcription elongation factor (P-TEFb) with CDK9. The CyclinT1/2 and CDK9 complex (P-TEFb) can phosphorylate the serine 2 of the carboxyl-terminal domain (CTD) of RNA polymerase II and activate this enzyme, and promote transcription elongation [18,19,29,30]. It is noteworthy that Ku80, another component of the DNA-PK complex, can also bind to the RNA polymerase □ elongation complex (not initiation complex) [31]. Although the authors demonstrated that the binding of Ku80 with Pol □ was not dependant on DNA-PKcs [31], it was evident that there is a close relationship between DNA-PK and the gene transcription complex. Therefore, interaction of DNA-PKcs with Cyclin T2 (or P-TEFb) suggests that DNA-PK is closely associated with the transcriptional "machinery" both physically and physiologically. The binding of these two molecules might have two biological functions: regulation of both gene transcription and the transcription-coupled repair (TCR) mechanism. Since RNA Pol II has already been verified to play an important role in the initialization of TCR [18,19], we postulate that the involvement of DNA-PKcs in TCR might be mediated through its interaction with Cyclin T2/CDK9/RNA Pol II.

## Conclusion

A new method of TCR assay based on the strand-specific-PCR (SS-PCR) was developed. This new method has been validated through confirming the decreased TCR efficiency of silenced c-myc gene in DMSO-treated HL60 cells and the *DHFR *gene in CSB-depleted HeLa cells after UV irradiation. Our data suggest that DNA-PKcs plays a role in the TCR pathway of UV-damaged DNA. The interaction of DNA-PKcs and cyclin T2 was revealed. Therefore, we assume that DNA-PKcs possibly functions via associating with the P-TEFb complex (cyclinT2/CDK9) to modulate the activity of RNA Pol II, which has already been identified as a key molecule recognizing and initializing TCR.

## Methods

### Cell Culture and UV irradiation

HeLa-H1, HeLa^siRNA-107 ^and HeLa-NC cells were derived from HeLa cells by stably transfecting the vector expressing the specific siRNA targeting the catalytic motif of the DNA-PKcs gene (nucleotides 11637~11655, H1) [15], targeting the CSB gene (nucleotides 1507-1525 of CSB, gi: 4557564) [32], and using a control siRNA construct. Parental HeLa and the derivative cells were maintained in Dulbecco modified Eagle medium (DMEM) containing 10% fetal bovine serum, 100 U/ml penicillin and 100 μg/ml streptomycin in a humidified chamber at 37°C in 5% CO_2_. HL60 cells were maintained in RPMI1640 medium containing 10% fetal bovine serum, 100 U/ml penicillin and 100 μg/ml streptomycin in a humidified chamber at 37°C in 5% CO_2_. Cells were UV-irradiated at room temperature after washing twice and replacing the medium with cold sterile phosphate buffered saline (PBS). The SpectroLinker XL-1000 UV Crosslinker (Spectronics Corporation, Westbury, NY) was used as Ultraviolet light (UV) source, which consists of five 8-Watt UV-B tubes emitting at 312 nm. After UV irradiation, the cells were collected immediately or further cultured with growth medium for a given time of DNA repair, or subjected to the assay of cell growth, survival and DNA repair.

### Cell growth and survival

After exposure to 312 nm UV light radiation at room temperature, HeLa-H1 and HeLa -NC cells (5 × 10^3^/well) were seeded in 24-well culture plates. The cell numbers from three wells were counted every day after plating for each group. Four independent experiments were performed and the mean values were used to derive the growth curve.

To assay the effect of UV radiation on cellular colony-forming ability (cell survival), an appropriate number of cells (3 × 10^2 ^to 1 × 10^4^) were plated into 60-mm petri dishes in triplicate. After the cells attached onto the plates, the culture medium was removed and cells were immediately exposed to UV radiation and corresponding controls were sham irradiated. Fresh growth medium was added after irradiation and the cells were continuously cultured for two weeks. Cells were fixed with methanol, stained with Giemsa solution, and colonies (consisting of more than 50 cells) were counted. Three independent experiments were performed for this clonogenic assay. The data of surviving colonies were normalized with the plating cell numbers and used to plot survival curves.

### Differentiation of HL60 cells induced by DMSO

The proto-oncogene c-myc gene is silenced in HL60 cells when the cells are induced to differentiate with DMSO[21]. HL60 cells were subcultured in flasks, DMSO was added to a final concentration (v/v) of 0.5% or 1% 24 h later, and cells were continuously cultured for different periods of time (12, 24, 48, 72 and 96 h). After this, the cells were harvested and subjected to immunoblotting analysis of c-myc expression and TCR assay.

### Preparation of genomic DNA samples for DNA repair detection

1) Genomic DNA extraction: Adherent cells (HeLa-NC, HeLa-H1, HeLa^siRNA-107^) were seeded in 60-mm Petri dishes. After growing to 70~80% confluence, the cells were exposed to UV radiation (10 J/m^2^), then collected at different time points (0, 2, 4, 8 and 24 h) after irradiation. The genomic DNA was extracted according to the Genomic DNA extraction kit manual (Qiagen, Hilden, Germany). The suspension of HL60 cells was treated with DMSO (1%, v/v) for 72 h to induce differentiation, then collected by centrifugation, and resuspended with PBS solution. A tiled monolayer of cells was made by dropping the cell suspension into 60-mm dishes. Then the cells were exposed to 10 J/m^2 ^UV radiation. After UV irradiation, cells were continuously cultured for different times (0, 4, 8, 24, 48 h) and harvested to extract genomic DNA as described above.

2) Digestion of genomic DNA with T4 endonuclease V: As T4 endonuclease V (Endo V) has N-glycosylase and apurinic/apyrimidinic lyase (AP lyase) activities; it can locate and bind to pyrimidine dimers in dsDNA induced by UV radiation. It cleaves the N-glycosylic bond of the 5' pyrimidine of the dimer and breaks the phosphodiester bond 3', which results in an abasic site. Therefore, the genomic DNA with residual pyrimidine dimers can be fragmented into small pieces by T4 Endo V. A reaction solution was prepared as follows: 2 μl 10 × buffer of T4 EndoV, 2 μg genomic DNA, 2 U T4 Endo V (Epicentre Biotechnologies, Madison, WI, USA), supplemented with distilled water to a final volume of 20 μl. The endonuclease digestion reaction was conducted at 37°C for 1 h, and then part of the reaction product was directly mixed with 6 × alkaline loading buffer and subjected to alkaline agarose gel electrophoresis of the global genomic nucleotide excision repair (ggNER) assay. The rest of the reaction product was eluted and precipitated with ethanol for the strand-specific PCR amplification of the transcription-coupled repair (TCR) assay. It is important to purify the T4 EndoV-digested DNA with QIAquick PCR Purification Kit (QIAGEN Gmbh, Hilden, Germany), for insuring the specific amplification of the TCR assay.

### Detection of ggNER of UV-induced DNA damage by alkaline electrophoresis[33,34]

The genomic DNA samples digested with or without T4 Endo V were mixed with 6 × alkaline loading buffer and subjected to alkaline agarose gel electrophoresis separation for 4-6 h at 40 volts at 20~25°C, then stained with ethidium bromide for 30-60 min, and finally visualized and analyzed with an electrophoresis gel formatter imaging system.

### TCR Detection of UV-induced DNA damage by strand-specific PCR

The *DHFR *and c-myc genes were selected for the assay of TCR as previously reported[20,21]. In order to compare the efficiency of DNA damage repair between the active transcribed-strand and the untranscribed-strand of the *DHFR *or c-myc genes, we adopted the strategy of single strand-specific PCR to amplify both the active transcribed- and non transcribed-strands separately. The DNA strand can be fragmented by T4 Endo V when there are residual pyrimidine dimers on it. Therefore, the PCR efficiency of the T4 Endo V digested active transcribed- or untranscribed-stranding represents the TCR capability. This strategy of DNA single strand-specific PCR detection was partially derived from the idea of the suppression subtractive hybridization PCR method [23]. The detail of this strategy is described in the results section (Figure 3A).

siRNA molecules and the PCR primers for all amplifications were synthesized by Shanghai GeneChem Co., Ltd (Shanghai, China). The primers sequences and PCR conditions are listed in Table 1.

1) The first-strand synthesis of the transcribed strand or non-transcribed strand: Four primers were designed and used to synthesize the first-strand of the transcribed strand (DHFR_UP-TSP1, c-myc_UP-TSP1) and the non-transcribed strand (DHFR_UP-NTSP1, c-myc_UP-NTSP1) for the *DHFR *and c-myc genes, respectively (Table 1). These primers are composed of an up-stream adaptor universal primer (UP), which shows no homology to the mammalian genomic DNA, and a down-stream annealing primer, which specifically targets the transcribed strand or untranscribed strand of the *DHFR *or c-myc genes. The adaptor universal primer sequence is added into the newly synthesized first-strand, which is then used for the purpose of strand-specific PCR. The first-strand synthesis mixture includes: Endo V digested or non-digested DNA sample 0.5 μg, 10 × PCR buffer 1 μl, 0.25 μM dNTP, 0.2 μM first-strand synthesis primer, 2.5 U Taq enzyme, supplemented with distilled H_2_O to a final volume of 10 μl. After the digestion using the conditions shown in Table 1, the mixture was purified with QIAquick PCR Purification Kit to eliminate the free primer, and subjected to the following strand-specific PCR amplification.

2) The strand-specific PCR amplification of the transcribed strand or non-transcribed strand: For the strand-specific PCR, the newly synthesized first-strand is used as template, the inserted adaptor universal primer is used as the upstream primer (UP), and the primers targeting a downstream sequence of the newly synthesized first-strand of the transcribed strand or the non-transcribed strand are used as downstream primers (Table 1). Although the primary genomic DNA could also be synthesized during this PCR reaction, it would be by linear amplification and not logarithmic amplification because the adaptor upstream primer UP cannot anneal with primary genomic DNA. Therefore, the transcribed strand and non-transcribed strand can be separately synthesized in the manner of logarithmic amplification. The PCR mixture includes: the synthesized first-strand DNA sample 100 ng, 10 × PCR buffer 1 μl, 0.25 μM dNTP, 0.2 μM upstream primer UP, 0.2 μM downstream primer DHFR_TSP2 or DHFR_NTSP2/c-myc_TSP2/c-myc_NTSP2, 2.5 U Taq enzyme, supplemented with distilled H_2_O to a final volume of 10 μl. The PCR reactions used the conditions shown in Table 1. The PCR products were visualized and analyzed using an electrophoresis gel formatter imaging system.

Amplification of a β-actin fragment without the TT sequence (no TT dimer formation after UV irradiation) was used as internal template reference and for the PCR conditions control.

### Co-immunoprecipitation (CoIP) and protein identification

Co-immunoprecipitation was performed using the Immunoprecipitation Kit (Protein A/G, Roche Molecular Biochemicals) according to the manufacturer's instructions. Briefly, HeLa cells were washed twice with ice-cold PBS and collected by centrifugation. The cell pellets were resuspended in pre-chilled lysis buffer (50 mM Tris-HCl, pH 7.5, 150 mM NaCl, 1% Nonidet P40, 0.5% sodium deoxycholate and a certain amount of the complete tablet provided by the Kit) and homogenized. The supernatants were collected by centrifugation at 12 000 × g for 10 min at 4°C to remove debris and then subjected to immunoprecipitation. After preclearing with protein A/G-agarose, the supernatants were incubated for 3 h with 2 μg of anti-DNA-PKcs antibody or anti-Cyclin T antibody at 4°C followed by overnight incubation with protein A/G-agarose at 4°C. The immunoprecipitates were collected by centrifugation, washed twice with washing buffer 1 (50 mM Tris-HCl, pH 7.5, 500 mM NaCl, 1% Nonidet P40 and 0.05% sodium deoxycholate) and then once with washing buffer 2 (10 mM Tris-HCl, pH 7.5, 0.1% Nonidet P40 and 0.05% sodium deoxycholate). A part of the immunoprecipitate products of anti-DNA-PKcs antibody was separated on 4% - 15% graded SDS-PAGE and stained with Coomassie bright blue dye. The visible bands were excised and subjected to the identification of peptide mass fingerprint analysis using the MALDI-TOF-Mass Spectrometric Analysis and software of SWISS-PROT/TEMBL database, which was carried out by the National Center of Biomedical Analysis. The remaining part of the immunoprecipitate product of the anti-DNA-PKcs antibody was denatured by heating to 100°C for 3 min in gel-loading buffer and centrifuged at 12 000 × g for 20 s to remove the protein A/G-agarose. The denatured proteins were resolved by 8% SDS-PAGE and subjected to immunoblotting analysis with an anti-DNA-PKcs antibody.

### Immunoblotting analysis

The cells were harvested and washed twice with ice-cold PBS. Cell pellets were incubated with lysis buffer (50 mmol/L Tris-HCL, pH 7.5, 1% Nonidet P40, 0.5% Sodium deoxycholate, 150 mmol/L NaCl, 1 piece of Protease inhibitor cocktail tablet in 50 ml solution) and the total protein was isolated. Proteins (50 μg) were resolved by sodium dodecyl sulfate polyacrylamide gel electrophoresis (SDS-PAGE, 8%) and then transferred onto the polyvinylidene difluoride (PVDF) membrane for immunoblotting. The immunohybridization was performed with anti-c-myc antibody (Santa Cruz Biotechnology, CA, USA), anti-CSB antibody (Santa Cruz Biotechnology) or anti-cyclin T2 antibody (Santa Cruz Biotechnology, CA, USA). The secondary antibody IgG-HRP was purchased from Zhongshan Co. (Beijing, China). Protein expression levels were detected using the luminal analysis reagents (Santa Cruz Biotechnology, CA, USA) according to the manufacturer's instructions.

## Competing interests

The authors declare that they have no competing interests.

## Authors' contributions

JA and TYY carried out most of the study and participated in its discussion. YCH participated in the experiments of the strand-specific PCR measurement of *DHFR *gene after UV radiation exposure. FL constructed the cell model of depressed *CSB *gene mediated by siRNA strategy. JFS identified the interaction of DNA-PKcs and cyclin T2. YW participated in the establishment of DNA-PKcs depleted HeLa-H1 cells. QZX participated in the work design and data discussion. DCW jointly conceived of the study and coordination. PKZ jointly conceived of the study, and coordination, participated in its design and drafted the manuscript. All authors read and approved the final manuscript.
